# Heterologous expression and antimicrobial potential of class II bacteriocins

**DOI:** 10.1080/19490976.2024.2369338

**Published:** 2024-06-20

**Authors:** Carola Elisa Heesemann Rosenkilde, Ditte Olsen Lützhøft, Ruben Vazquez-Uribe, Morten Otto Alexander Sommer

**Affiliations:** The Novo Nordisk Foundation Center for Biosustainability, Technical University of Denmark, Kongens Lyngby, Denmark

**Keywords:** Heterologous expression, bacteriocins, gut microbiota, antimicrobial peptides, microbiome modulation

## Abstract

Gut bacteria are known to produce bacteriocins to inhibit the growth of other bacteria. Consequently, bacteriocins have attracted increased attention as potential microbiome-editing tools. In this study we examine the inhibitory spectrum of 75 class II bacteriocins against 48 representative gut microbiota species. The bacteriocins were heterologously expressed in *Escherichia coli* and evaluated *in vitro, ex vivo* and *in vivo. In vitro* assays revealed 22 bacteriocins to inhibit at least one species and showed selective inhibition patterns against species implicated in certain disorders and diseases. Three bacteriocins were selected for *ex vivo* assessment on mouse feces. Based on 16S rRNA sequencing of the cultivated feces we showed that the two bacteriocins: Actifencin (#13) and Bacteroidetocin A (#22) selectively inhibited the growth of *Lactobacillus* and *Bacteroides*, respectively. The probiotic: *E. coli* Nissle 1917 was engineered to express these two bacteriocins in mice. However, the selective inhibitory patterns found in the *in vitro* and *ex vivo* experiments could not be observed *in vivo*. Our study describes a methodology for heterologous high throughput bacteriocin expression and screening and elucidates the inhibitory patterns of class II bacteriocins on the gut microbiota.

## Introduction

The increasing concerns surrounding food safety, antibiotic resistance, and the intricate relationship between the gut microbiota and human health have prompted extensive research on bacteriocins. These naturally occurring antimicrobial peptides, produced by a diverse range of bacteria, have garnered interest for their ability to target closely related species.^1^ Bacteriocins exhibit both narrow and broad-spectrum activities and possess desirable characteristics such as nanomolar range efficacy, diverse modes of action, and high specificity compared to traditional antibiotics.^2–4^ Additionally, their proteinaceous nature allows for engineering to enhance specificity and stability.^5,6^

Based on structural and functional attributes, bacteriocins are classified into three major types.^7^ Among these types, class II bacteriocins, predominantly derived from Lactic Acid Bacteria (LAB), have been extensively studied *in vitro* for their potential as bio-preservatives in food, particularly against undesirable pathogens such as *Listeria* spp.^8,9^ Apart from their role in food preservation, class II bacteriocins have also been explored as alternative antibiotics for medical and veterinary applications, including combating *C. difficile* infections.^4,10,11^ *In vivo* investigations of class II bacteriocins have demonstrated their ability to eliminate target bacterial strains without significantly affecting the overall microbiome composition, often employing the natural bacteriocin producer strain.^8,12,13^ However, limited studies have examined the impact of bacteriocins on the overall composition of the gut microbiota, and species that are commensals as well as implicated in certain health disorders.

Understanding the mechanisms underlying bacteriocin-mediated microbiota modulation is crucial for their effective utilization as antimicrobial agents.^14^ Systematic and high-throughput studies of bacteriocins can be challenging when relying solely on natural bacteriocin producers due to low production levels and complex growth conditions. Therefore, utilizing heterologous hosts offers advantages for *in vitro* screening and controlled *in vivo* delivery of bacteriocins. This approach circumvents the need for specific regulatory conditions in the native producer and facilitates large-scale production for food and therapeutic applications.^15^ *Escherichia coli* (*E. coli*), extensively characterized as a production host for bacteriocins and proteins, is commonly employed due to its well-established features.^15,16^

A typical bacteriocin gene cluster comprises a leader peptide, bacteriocin gene, immunity gene, modifier gene, and a secretion machinery such as an ABC transporter.^17^ However, for class II bacteriocins, which possess a simpler structure, heterologous expression solely using the bacteriocin gene itself is feasible. By utilizing a tight and inducible expression system, the need for an innate immunity gene can often be eliminated.^18^ Moreover, employing a native signal peptide for *E. coli*, such as OmpA, eliminate the need for a bacteriocins specific leader peptide for transport, as the bacteriocin can be secreted using the native *E. coli* secretion system^19^ Recently, Mortzfeld et al.^20^ expressed Microcin MccI47 from the native *E. coli* plasmid pMUT2. The study showed that heterologously expressed Microcin MccI47 were able to inhibit the growth *in vivo* against *K. pneumonia*, thereby emphasizing the potential of heterologously expressed class II bacteriocins as potential future antibiotics, but also to understand their role in modification of the native gut microbiota.

Here, we describe a heterologous expression platform for high throughput expression and screening of 75 class II bacteriocins on a collection of 48 representative gut microbiota strains. Our study encompasses common gut species including relevant pathogenic species and probiotic bacteria, as well as species implicated not only as part of the core microbiota but also as related to health disorders. Additionally, we apply the heterologous expression platform to evaluate the effect of two broad spectrum bacteriocins on a complex microbiota both *ex vivo* and *in vivo*. The findings from this research provide valuable insights into the target range of class II bacteriocins, thereby contributing to their potential future application.

## Materials and methods

### Selection of bacteriocin genes

To obtain a non-redundant list of bacteriocin sequences, all class II bacteriocins available in the BAGEL3^21^ and Bactibase^22^ databases were downloaded, resulting in a total of 233 sequences (date of download: 5^th^ of February 2020). This list was filtered based on the inclusion criteria: being non-redundant (143 sequences), having all amino acids verified (no X amino acids in the sequence) (102 sequences), being one compartment (The bacteriocin alone has an effect as opposed to two or more bacteriocins working together to create an effect) (95 sequences), and having a 100% sequence similarity with a protein in a blast-search (https://blast.ncbi.nlm.nih.gov/Blast.cgi) (this was to verify the reliability of this exact sequence). This led to a total of 79 sequences. Amino acid sequences and references can be found in Supplementary Table S1. The two bacteriocins: Actifencin^23^ and Bacteroidetocin A^24^ was added to the collection, due to their status as being similar to class II bacteriocins but having a different target spectrum. From this list 75 bacteriocins could be successfully cloned into *E. coli* as verified by Sanger sequencing with insert primers. The DNA sequences was codon optimized for production in *E. coli* K-12 from IDT online codon optimization tool (https://eu.idtdna.com/CodonOpt).

### In vitro screening

#### Selection of gut strains to build the representative gut strain catalogue

The gut strains were selected based on a literature search of the following studies examining species to be considered part of the core human microbiota.^25–29^ Furthermore, we used the studies of^26,29^ to identify the species ability to grow in the rich medium mGAM, since this would allow for high throughput screening of all included gut species. mGAM has shown to be the best selective media for isolating gut bacteria.^30^ This led to a total of 59 species. Based on their presence and growth capability in mGAM as well as their availability from www.dsmz.de. and www.atcc.org the final list was reduced to 43 species. To this list we included two *E. coli* strains: *E. coli* Nissle 1917 and *E. coli* K-12 from an internal lab collection, *L. inoccua DSM 20,649*, *L. mali DSM 20,444*, *L. delbrueckii subsp. Bulgaricus* DSM 20,074, *L. amylovorus* DSM 16,698, *E. faecalis* ATCC 19,433, *K. pneumoniae* DSM 681, and a *C. difficile* isolate 7–6011209 isolated from fecal samples derived from an internal lab collection to reach a total of 52 strains. All strains were ordered from DSMZ or ATCC unless otherwise stated. From this final collection, four did not grow in mGAM media, reducing us to the 48 strains finally used in our study. Supplementary Table S2 contains a complete list of the microorganisms used in this study, as well as their prevalence in the human gut based on the study by Nielsen et al..^31^

#### *Plasmid and strain construction for expression in* E. coli *BL21-AI*

Primers and gBlocks were ordered from Integrated DNA Technologies (IDT). gBlocks containing the bacteriocin sequences were ordered from Twist Bioscience. Sequenced can be found in Supplementary table S2. List of bacteriocin sequences can be found in Supplementary Table S1. Native *E. coli* Nissle plasmid pMUT1 was used as the vector to produce the bacteriocins. This plasmid was originally cured and used in the study by Armetta et al.^32^ and kindly donated to us by the authors. The plasmid differs from the original pMUT1 plasmid with the presence of a kanamycin resistance gene, as well as a Hok/Sok toxin-antitoxin gene cluster. The native signal sequence for *E. coli* OmpA^33^ was placed immediately in front of the bacteriocin gene. A T7 promoter sequence was used to facilitate arabinose induction from the BL21-AI strain. All plasmid assemblies were conducted with Gibson assembly^34^ and transformed into *Escherichia coli* One Shot TOP10 (Thermo Fisher Scientific) via electroporation. Cells were recovered in SOC +0.5 ml 1 M Mg2Cl2 + 2 ml 1 M glucose, for 1 h at 37°C with shake, then plated on LB agar plates containing 50ug/ml kanamycin (Roth Art-Nr: T832.3) and incubated at 37°C ON. All *E. coli* were grown in lysogeny broth (LB) (Sigma Aldrich) unless something else is specified. Colony-PCR using OneTaq (Thermo Scientific^™^) confirmed the plasmid assembly. PCR product was Sanger Sequenced using Eurofinsgenomics. Plasmids were extracted using plasmid extraction kit (Machery-Nagel – Nucleospin plasmid easy pure − 740725.250) and transformed into Invitrogen^TM^ BL21-AI^TM^ Oneshot ® Chemically Competent *E. coli* according to suppliers’ protocol (Thermo Fisher Scientific 11,540,146) and selected on LB-kanamycin plates. Colony-PCR using OneTaq was performed again to verify integration into BL21-AI using Sanger sequencing.

#### Evaluations of optimal expression conditions for the bacteriocins

Optimal growth conditions were examined using the target strain *L. mali* DSM 20,444 using 8 different bacteriocins. The following growth conditions were tested: incubation temperatures of the plates (from the time BL21-AI was spotted on the plates to the end of the study) at 25°C, 30°C, or 37°C, preculture overnight growth of BL21-AI in either mGAM of 2-YT, 6 h of growth versus 18 h of growth of the spotted BL21-AI culture on the plate before pouring the top agar, 0.2% vs 1% of arabinose concentration in the culture plates. The inhibition zones of the bacteriocins were used to evaluate the optimal growth conditions (which were very comparable through all the groups). The following protocol produced the largest inhibition zones for all bacteriocins tested.

#### Expression of bacteriocins for spot assay

All bacteriocin expression for spot assays was performed in the following manner unless something else is stated. The bacteriocin producer was streaked on LB-kanamycin plates from a −80 cryo-stock and incubated at 37°C ~14–18 h. One colony was inoculated into 2-YT media containing 50 μg/ml of kanamycin and incubated with shake for ~14–18 h. Cultures were spun down and resuspended in PBS, then spun down again and resuspend in 2-YT to make sure all kanamycin was gone from the media. 10 μl of the culture was spotted on mGAM square plates containing 1% arabinose (for induction of the bacteriocin gene via the T7 promoter). Plates were placed in aerobic conditions at 37°C for ~20 h. After 20 h the plates that were used to screen anaerobic strains were transferred to ANO boxes and incubated further at 37°C ON (~20 h) to pre-reduce the plates. Plates that were used to screen aero-tolerant strains were poured with top agar after the initial 20 h of incubation. As a negative control was used BL21-AI with a pMUT1 plasmid containing no bacteriocin gene.

#### Cultivation of target strains for spot assay

Target anaerobic gut strains were grown in mGAM where possible. Aerobic Lactic acid bacteria were grown in MRS, *E. faecalis* and *L. innocua* were grown in BHI. Anaerobic strains were streaked from −80°C cryo-stocks under anaerobic conditions (Whitley A95 Workstation – Don Whitley Scientific); gas mixture, 95% N_2_ and 5% H_2._ Lactic acid strains were streaked from −80°C in aerobic conditions on MRS and transferred to anerobic boxes. Strains growing on BHI were streaked aerobically and incubated aerobically. All strains were incubated at 37°C ~18 h. One colony was used to inoculate 1 ml of the respective media and incubated for additionally 18–20 h prior to the spot assay. Strains with Biosafety level 2 were handled in the same manner, except that a Coy Laboratory Products Vinyl; gas mixture, 95% N_2_ and 5% H_2_ was used to make anaerobic conditions.

#### Overlay agar for spot assay

For each square plate (Thermo fisher scientific omnitray w/lid Non treated sterile #264728) in total of 12 ml pre-reduced top agar was used (0.5% agar) (Milipore # 69964) +100 μl of the target strain adjusted to OD ~ 0.5. Aerobic strains: Strains were mixed with top agar and poured over the plates. The plates were transferred to anaerobic boxes and incubated for 1–2 d at 37°C. Anerobic strains: Agar plates with spots of BL21-AI bacteriocin producers and the liquid cultures containing the target strains, were transferred from anaerobic conditions into an aerobic laf bench. Top agar (~40°C) was mixed with each of the target strains and poured over the respective plates and left to dry for ~5–10 min before being transferred back to the anaerobic chamber and incubated in anaerobic boxes at 37°C for 1–2 d. After 1 and 2 d of growth the plates were checked for inhibition zones. All strains were tested in at least duplicates. Halosize in mm can be found in Supplementary Table S4.

#### Data analysis

Heatmap with inhibited species against the bacteriocins with at least 1 target strain was generated using R (phyloseq). NCBI common taxonomy tree^35^ was used to build a phylogenetic tree of the target species, and the heatmap was ordered according to that. Clustal Omega^36^ was used to create a multiple alignment of the protein sequences of the bacteriocins, and the heatmap was sorted according to that.

### Ex vivo screening study

#### Growth media preparation

The following plates were used in the study: MRS containing 25% of *E. coli* BL21-AI supernatant from either BL21-pMUT0-no-bacteriocin, BL21-pMUT12-ubericinA, BL21-pMUT13-actifencin, to select for a variety of LAB strains. mGAM agar plates containing 25% of supernatant from BL21-pMUT0-no-bacteriocin, BL21-pMUT12-ubericinA, BL21-pMUT13-actifencin, BL21-pMUT22-bacteriodetocinA to select for gut strains in general. Furthermore, we used mGAM plates with 25% water to examine the effect of potential selection differences between the water control and the BL21-pMUT0-no-bacteriocin control. mGAM-vancomycin (5ug/ml)-kanamycin (50ug/ml) containing 25% of supernatant from BL21-pMUT0-no-bacteriocin, BL21-pMUT22-bacteroidetocinA, to select for a variety of *Bacteroides* strains. Supernatant for creating the agar plates were produced in the following manner: BL21-AI cultures were streaked on LB-kanamycin (50ug/ml) agar plates from −80°C cryostocks and incubated at 37°C for ~20 h. One colony was inoculated into 5 ml 2YT-kanamycin (50ug/ml) and incubated with shake ~18 h at 37°C. Cultures were diluted 1:100 into fresh 200 ml 2YT media in 1 L shake flasks – without antibiotics and incubated with shake at 37°C until OD reaches 0.4–0.5. Cultures were then induced with 1% arabinose and incubated with shake for 6 h at 30°C. After 6 h the cultures were centrifuged for 5 min in 50 ml falcon tubes at 4500 × G at 4°C, 1 tablet of protease inhibitor (Roche – cOmplete ULTRA Tablets, Mini, EDTA-free #05892791001) was added to every 50 ml of supernatant. The supernatant was sterile filtered with 200 ml 0.22um filter cups (Biofil – FPV213500). Supernatant was immediately used to make agar plates by mixing with 60°C freshly prepared 1.25X concentrated media. mGAM and mGAM-vancomycin-kanamycin plates were moved to anaerobic environment for prereduction for 24 h. MRS plates were stored aerobically at 5°C.

#### Feces collection

Feces from 3 male C57BL/6nTAC mice that had received CHOW diet, and no antibiotics was collected and transferred to anaerobic environment within ~10 min. Pellets were resuspended in 1 ml pre-reduced 1% PBS. After resuspension the tubes were left for ~20 min to allow sediment to form. 2 × 200 μl of the samples were transferred to 1.5 ml Eppendorf tubes and centrifuged at 10.000 × G for 10 min. Supernatant was removed and the pellet was stored at −20°C, for sequencing. 100 μl of the fecal samples was used to make serial dilutions in 1% PBS down to 10^−8^. 100 μl of the three independent biological replicates were plated of the dilutions 10^−4^ −10^−8^ on mGAM and mGAM-vancomycin-kanamycin plates. Plates were incubated anaerobically at 37°C. For the MRS plates, selecting for lactic acid bacteria, the dillutions 10^−3^−10^−7^ were used. MRS plates were incubated aerobically, to allow further selection for lactic acid strains, at 37°C. Colonies were counted every day for 5 d – until no new colonies appeared on the plates. Two dilutions, consisting of the countable dilutions (between 25 and 250 colonies) were chosen for sequencing. None of the plate dilutions utilized represented a situation where a lawn was formed or the plate would not be able to be counted. 2 ml 1XPBS where administered onto the plates and a spatula was used to mix the colonies on the plates. ~1.2 ml was collected in 1.5 ml eppendorph tubes, and centrifuged 15 min 12000XG. Supernatant was removed at pellets were frozen at −20 until DNA extraction. The pelleted samples were then extracted as individual dilutions using ZymoBiomics DNA Kit (D4300- zymoresearch).

#### DNA preparation for and 16S rRNA MiSeq sequencing

All DNA samples were prepared for sequencing using the following protocol: “16S Metagenomic Sequencing Library Preparation^37^”. In short: Extracted DNA was diluted to 5 ng/ml for all samples to keep quantities constant for the amplification step. PCR was performed using KAPA PCR Master Mix (Roche) and tagged Illumina primers (10 mM concentration) in 25 ml reactions targeting the hypervariable V3-V4 (341F − 785 R) region (primer are listed in Supplementary Table S3). Illumina overhangs (100 mM concentration) were attached in a second PCR reaction by combining barcoded samples in equal amounts as template for amplifying multiple 50 ml reactions. Thermocycling conditions for both PCR steps were as follows, except 25 cycles in the first PCR and 8 cycles in the second PCR: initial denaturation 95°C for 3 min, followed by 25 or 8 cycles of 95°C for 30 s, 55°C for 30 s and 72°C for 30 s, and a final elongation at 72°C for 10 min. PCR product sizes were confirmed at each step and the final PCR product was purified using AMPure XP bead (Beckman coulter). The samples were measured with qubit and normalized to 10 nM/μl (diluted in Tris-HCL pH:8.5), then run on Agilent 2100 Bioanalyzer (Covaris) to verify the size of the fragments, and sequenced on an Illumina MiSeq system.

#### Sequence quality control and processing

Fastq files were downloaded from Basespace.illumina.com. Qiime2 was used to process the fastq files to count matrices, followed by downstream data analysis using R. The following tutorial was used to perform the analysis using Qiime2.^38^ In short: fastq files were imported into qiime2 as paired end with input phred33. Quality filtering, chimera checking, and paired- end read joining of the sequence data was perform with DADA2^39^ through the q2-dada2 plugin.

#### For the ex vivo study

Reads were truncated when the quality score became approximately below 25 (forward reads at 285 bp, reverse reads at 240 bp). Reads were filtered from each sample (between 55.49% and 89.85% with a mean of 85.32% - reads per sample were between 4621 and 633,583 with a mean of 89,248 reads).

#### For the in vivo study

Reads were truncated when the quality score became approximately below 25 (forward reads at 260 bp, reverse reads at 240 bp). Reads were filtered from each sample with a mean of 57% - reads per sample were between 401 and 104.077 with a mean of 70.255 reads).

#### For both the ex vivo and in vivo study

A feature table and feature data were generated using the command “qiime feature-table” describing the ASVs that were observed in each sample and how many times it was observed. To assign taxonomic information to the ASV sequences a trained classifier for the V3-V4 region based on the “SILVA release_139 nr99” SSU database, which uses 99% similarity to assign species to an ASV. The classifier was downloaded from Github: https://github.com/anweshmaile/silva-138_classifiers. The command “qiime feature-classifier” was used for this analysis, outputting a count matrix used for further processing in R using the phyloseq package. In R the further data processing was handled. ASVs were removed if they had less than 2 counts in at least 10% of samples. This reduced ASVs from 4541 to 332 taxa in the *ex vivo* study and removing ASVs with less than 2 counts in 5% of samples reduced the number of taxa from 4600 to 2293 in the *in vivo* study. Rarefaction curves were made for both studies with curves showing max species at ~2000 species in both cases, therefore rarefaction was performed using 2000 species per sample. This removed five samples from the *ex vivo* data, to leave in total 76 samples, and one sample in the *in vivo* data to leave in total 71 samples. Beta-diversity was examined using Bray-Curtis method quantifying the difference between the overall taxonomic composition between samples.

### In vivo study

#### *Plasmid and strain construction for expression in* E. coli *Nissle*

Primers, promoters and sequences are listed in the Supplementary Table S3. Primers and geneblocks were ordered from Integrated DNA Technologies (IDT). The probiotic strain used in this study, *EcN*_GFP-StrepR is a modified version of the wild-type *E. coli* Nissle 1917 (tradename Mutaflor, Ardeypharm, Germany) strain.^40^ The same plasmid: pMUT1-kanR-Hok/Sok as was used in the *in vitro* and *ex vivo* study was used in the *in vivo* study. *EcN*_GFP was used as production host using a strong constitutive promotor (#1.7 from the Schantzetta library^32^). The bacteriocins were secreted using the OmpA signal sequence.^33^ The ribosomal binding site (RBS) of the bacteriocin gene was measured with salislab.net.^41^ Strength was ~5000 compared to the RBS in the pMUT1 plasmid used for *in vitro* expression of bacteriocins which was ~10.000. The following plasmids were cloned and expressed in *EcN*_GFP: *EcN-*pMUT0-no-bacteriocin, *EcN-*pMUT12-ubericinA, *EcN-*pMUT13-actifencin, *EcN-*pMUT22-bacteroidetocinA, and *EcN-*pMUT52_Bacteriocin31. Cloning and transformation, plasmid evaluation and purification was performed similar to the plasmid construction for *E. coli* BL21-AI expression

#### Generation of competent EcN

*EcN*_GFP was made competent for electroporation in the following way: Culture was streaked from −80°C cryo-stock on LB-streptomycin (50 μg/ml) agar plates and incubated at 37°C for ~20 h. Then, one colony was used to inoculate 5 ml 2YT-streptomycin (50 μg/ml) and incubated at 37°C for ~20 h with shake. Cultures were diluted 1:100 and incubated at 37°C with shake until OD reached 0.3–0.5 (~2 h). When desired OD was reached cultures were placed on ice for 15 min, then centrifuged for 10 min at 4°C and 4500×G. Supernatant was removed and pellet was resuspended in 1 ml MQ water +10% glycerol (4C). Cultures were centrifuged for 3 min at 6500RPM at 4°C, this step was repeated three times. After the last wash cells were resuspended in 50 μl MQ water +10% glycerol (4°C). 1 μl of the purified plasmid was used for electroporation of the 50 μl competent *EcN*_GFP cells. Cells were recovered in 1 ml fortified SOC for 1 h at 37°C with shake, then plated on LB agar plates containing 50ug/ml kanamycin and incubated at 37°C for 14–18 h. Colony PCR and gel electrophoresis was used to identify clones. Plasmids were extracted and subjected to whole plasmid sequencing using plasmidsaurus.com. Growth rate of the clones was evaluated using a plate reader (Synergy H1 - Holm and Halby). Two clones of each of the four *EcN*_GFP strains were inoculated 1:100 in 3 replicates into 100 μl LB-kanamycin (50ug/ml) in a 96-well plate. Growth was followed for 24 h in a plate reader (continuous shake 37°C) (Synergy H1 - Holm and Halby). Data were extracted and doubling time was calculated using R.

#### Animal experiment: ethics

The animal experiment was conducted according to the Danish Animal Experiments Act on protection of animals used for scientific purpose (LBK 1107 from 02/07/2022) and Directive 2012/63/EU of the European Parliament. In addition, the protocol was licensed accordingly by the Animal Experimentation Committee under the Ministry of Food, Fishing, and Agriculture (license number 2020-15-0201–00405). The study was carried out in accordance with the ARRIVE guidelines.^42^ Animal study design: 24 male C57BL/6NTac (Taconic Biosciences, Lille Skensved, Danmark) mice aged 5 weeks went through 7 d of acclimatization before being divided into four groups (*n* = 6) based on weight stratification. Hereafter, the mice received 1 daily oral dosing of 100 µl either containing PBS, *E. Coli* Nissle pMUT-empty (CFU 10^11^), *E. coli* Nissle pMUT13_actifencin (CFU 10^11^) or *E. coli* Nissle pMUT22_bacteroidetocinA (CFU 10^11^). CFU was measured based on OD600 measurements and standard curves created by spotting different dilutions of the gavage. After 7 d the mice went through a washout period of 7 more days. Colonization was investigated by fecal sampling on days: 0, 1,2,3,4,5,6,7, 9, 11, and 14. The mice were co-housed 3 per cage in individually ventilated cages (IVC). All mice were housed at 22°C ±2°C, light cycle was 6 am to 6 pm, and the mice were given ad libitum access to water and chow diet (Safe Diets, A30). At the end of the study, the mice were euthanized by CO_2_ sedation and cervical dislocation. Content from small intestine, cecum and colon collected in 1 × PBS to be tested for colonization (CFU count) immediately after collection.

#### Colonization

The feces were collected in pre-weighed 2.0 mL Eppendorf tubes including 1 mL of 1× PBS. After fecal samples had been added to the tubes these were weighed again to determine the fecal weight. All sample preparation for assessing CFU numbers was kept on ice and followed the same practice. The fecal samples were homogenized by vortexed at ~2400 rpm for 20 min. The samples were then spun down at 100×G for 30 s, followed by a dilution series, where 5 μL of each dilution was plated on LB supplemented with 50 mg/ml kanamycin and 50 mg/ml streptomycin. (Sigma Aldrich). After 24-h CFU was determined by counting. Samples were then spun down for 20 min at 11,000 × G, supernatant was removed, and pellet was stored at −20°C until DNA samples were extracted using DNeasy powersoil HTP 96 kit (qiagen Cat. No. 12955–4). DNA preparation for MiSeq 16S rRNA amplicon sequencing and sequence quality control was performed in the same manner as described for the *ex vivo* samples.

#### Verification of EcN strains from mouse feces

2–3 Fecal pellets from mice at study day 6 from the Actifencin (#13) and Bacteroidetocin A (#22) groups were resuspended in 200 ml 1X PBS and serial diluted to 10^-3. 100 μl of each dilution was plated on LB-kanamycin plates and incubated for 20 h at 37°C. *EcN*_pMUT22_bacteroidetocinA plates were transferred to ANO boxes and placed at 5°C for 24 h before performing the spot assay. Four colonies from two replicate mouse feces of *EcN*_pMUT13_actifencin was re-streaked on LB plates containing no antibiotics and incubated for additional 20 h before performing the spot assay. Plates containing *EcN*_pMUT22_bacteroidetocinA was tested using the indicator strain *Bacteroides vulgatus* DSM 1447. *EcN*_pMUT13_actifencin was tested using the indicator strain: *Lactobacillus mali* DSM 20,444. Indicator strains were cultured similar to what is described in the *in vitro* spot assay section. After pouring the top agar on the respective plates these were incubated ANO in the case for *EcN*_pMUT22_bacteroidetocinA and AE in the case for *EcN*_pMUT13_actifencin at 37°C. After ~20 h the plates were inspected for inhibition zones. Nine colonies of each of the *EcN* strains were subjected to colony PCR to verify that no mutations had occurred in the promotor or gene region of the plasmid. PCR product was Sanger sequenced with eurofinsgenomics.

### Media and antibiotic concentrations used and antibiotic references

Fortified SOC medium recipe: 100 ml SOC +0.5 ml 1 M Mg2Cl2 + 2 ml 1 M glucose, Tween80: Sigma-Aldrich 102,578,383, kanamycin sulfate: Roth Art-Nr: T832.3 (concentration used: 50ug/ml), streptomycin sulfate salt: Sigma-Aldrich – Merck Life Science 9137, Vancomycin: Sigma-Aldrich – Merck Life Science −94747-1 G, arabinose: Sigma Aldrich – L-(+)-Arabinose W325501, MRS deMan, Rogosa, Sharpe media: Milipore # 69966, MRS deMan, Rogosa, Sharpe agar: Milipore # 69964, mGAM media and agar (modified Gifu Anaerobic Media – Nissui pharmaceutical CO.,LTD), BHI (Brain Heart Infusion, Merck #53286)

## Results

### *In silico identification of class II bacteriocin genes and cloning into* E. coli

233 class II bacteriocins was downloaded from the bacteriocin databases: Bagel3^21^ and Bactibase.^22^ The sequences were filtered based on inclusion criteria down to 75 sequences (List of included bacteriocins can be found in supplementary Table S1). Among the cloned bacteriocins, three originated from Gram-negative species, while the remaining 72 originated from Gram-positive species. The inclusion of the two peptides to the list of class II bacterioins: Actifencin and Bacteroidetocin A was motivated by their close relationship to class II bacteriocins as well as their distinctive characteristics: Actifencin shows striking similarity to eukaryotic defensins and exhibits broad spectrum activity,^23^ whereas Bacteroidetocin A is shown to target mainly members of the Bacteroidetes phylum.^24^ By including these we aimed to broaden our understanding of class II related bacteriocins.

**Figure 1.**
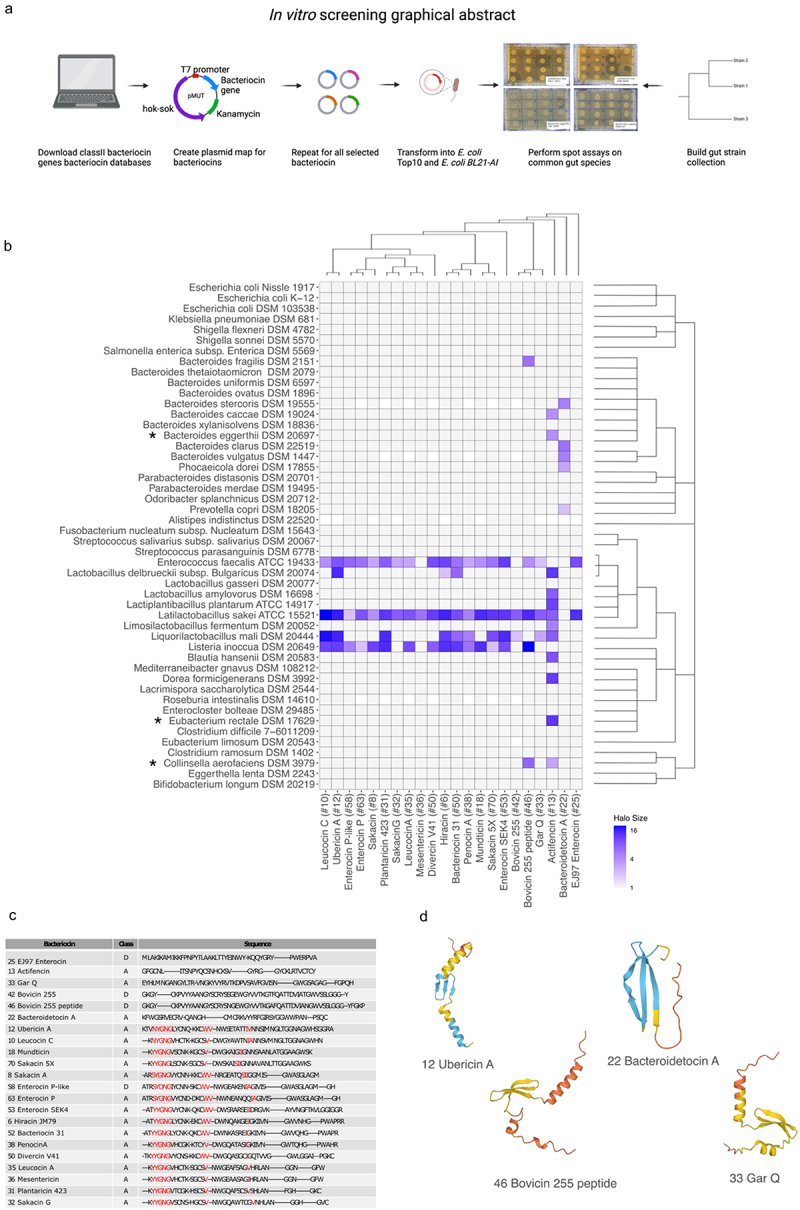
(a) Graphical abstract of the in vitro screening process. (b) Heatmap showing inhibited species based on spot assay of 48 common gut species. The heatmap is colored according to the halo-size of the in vitro assay. White tiles indicate NA values. The phylogenetic relationship of target strains and bacteriocin sequences are displayed to the right of the plot and on top of the plot, respectively. (*) indicates that this species has been implemented in diseases or disorders and not before has been characterized to be inhibited by this/these bacteriocins. (c) a multiple alignment for the functional bacteriocin sequences using Cobalt multiple alignment tool. Red AA indicates highly conserved regions. (d) Structures of the predicted bacteriocin sequences using alpha-fold,^43^ only the pro-peptide was used for the analysis. On the structure model, confidence is colored according to: Dark blue: Very high (pLDDT > 90) Light blue: Confident (90 > pLDDT > 70), Yellow: Low (70 > pLDDT > 50), Red: Very low (pLDDT < 50). The four structures shown are representative structures based on the sequence alignment. The lower helix on the three structures constitutes the leader sequence (except for Bacteroidetocin a (#22)).

The native bacteriocin producers spans 5 different phyla: *Bacillota* being the most prevalent, and 19 different genera with highest prevalence of *Lactobacillus* and *Enterococcus* species. The bacteriocin genes were inserted into the pMUT1 vector, native to *E. coli* Nissle.^32^ The insertion was performed downstream of the *E. coli* native OmpA signal sequence and fused with a *gfp* reporter gene. The expression of the inserted genes was controlled by an arabinose-inducible T7 promoter (Figure 1a). pMUT1 was chosen due to its high stability and retention over time.^44,45^

### Building the collection of representative gut microbiota strains

The gut strains included in this study were selected based on a literature search of the following studies examining species to be considered part of the core human microbiota.^25–29^ Furthermore, we used the studies of^26,29^ to identify the species ability to grow in the rich medium mGAM, since this would allow for high throughput screening of all included gut species. mGAM has shown to be the best selective media for isolating gut bacteria.^30^ From these studies a total of 48 strains were deemed suitable for inclusion in our representative gut microbiota collection (see Methods). Furthermore, we added to the list two *E. coli* species: *E. coli* Nissle 1917^40^ to test for inhibitory activity against the recombinant bacteriocin producer, and *E. coli* K-12 as a representative for the species *E. coli*. *Listeria inoccua* was added due to the general sensitivity of *Listeria* species toward class II bacteriocins.^46^ *Liquorilactobacillus mali* and *Enterococcus faecalis* ATCC 19433 was used as indicators for bacteriocin activity. *Lactobacillus delbrueckii* subsp. *bulgaricus* DSM 20074 and *Lactobacillus amylovorus* DSM 16698 was added due to their probiotic properties.^47,48^ *Klebsiella pneumoniae* DSM 681 and an isolate of *Clostridioides difficile* 7–6011209 was included due to their properties as opportunistic pathogens reaching a total of 56 strain. Of these strains, 48 grew on mGAM, MRS or BHI in our assays. The 48 strains consisted of 24 Gram-negative and 24 Gram-positive species, encompassing a range of phyla including *Bacillota* (11 species), *Firmicutes* (26 species), *Proteobacteria* (7 species), *Actinomycetes* (2 species), *Fusobacteria* (1 species), and *Actinobacteria* (1 species). Twenty strains were classified as Biosafety level 2 organisms. Several of the organisms under study exhibit dual characteristics: they are integral components of the normal microflora, as documented by Nielsen et al.,^31^ while also being implicated in various diseases and disorders. This dichotomy makes them ideal candidates for bacteriocin screening aiming to identify selective bacteriocins for novel, precise treatments that safeguard the balance of the gut ecosystem. For a comprehensive list of these strains, see Supplementary Table S2.

### In vitro screening of bacteriocins on gut microbiota strains

Class II bacteriocins were heterologously expressed in *E. coli* BL21-AI. The inhibition spectrum of each bacteriocin was assessed by conducting an overlay spot assay against each of the representative species of the gut microbiota. Inhibition was assessed through visual inspection of the plates and measurement of the inhibition halo size. A strain was considered inhibited if any inhibition zone was observed and exceeded the negative control (pMUT0). Among the 75 bacteriocins tested, 22 demonstrated inhibitory activity against at least one species in the assay and will henceforth be referred to as the inhibitory bacteriocins (Figure 1b). For the remaining 53 bacteriocins we cannot specifically say that these do not inhibit the tested species, as we have no proof of their actual production (lack of positive control).

Of the inhibitory bacteriocins, 21 originated from Gram-positive species and predominantly inhibited other Gram-positive species. However, Actifencin (#13), naturally produced by *Actinomyces ruminicola* DPC 7226, also displayed inhibition against the Gram-negative strain *Bacteroides eggerthii* DSM 20697. Notably, 5 out of the 24 Gram-negative strains were inhibited by at least one bacteriocin.

Bacteroidetocin A (#22), derived from the Gram-negative *Bacteroides vulgatus*, exclusively inhibited other Gram-negative species (4 species), including *B. vulgatus* itself (DSM 1447). The species most frequently inhibited by the bacteriocins were *Lactobacillus sakei* ATCC 15521 (21 bacteriocins), *Enterococcus faecalis* ATCC 19433 (18 bacteriocins), and *Listeria innocua* DSM 20649 (13 bacteriocins). Actifencin (#13) exhibited the highest inhibitory activity against the tested species, affecting 11 out of 48 strains.

To elucidate the observed variations in the inhibitory spectra among the bacteriocins, a multiple sequence alignment was performed using COBALT multiple alignment tool^49^ (Figure 1c). The analysis identified that the consensus sequence xYGNGV, known to be conserved among class IIa bacteriocins^24^ was present in a majority of the examined bacteriocins. In addition, these bacteriocins shared highly similar structural characteristics, comprising a helix, a beta-sheet, and in certain instances, a leader sequence that also adopted a helical conformation (Figure 1d).

The six bacteriocins exhibiting deviations from both the common structure and sequence patterns typical for class II bacteriocins: E97enterocin (#25) (class IId), Actifencin (#13) (similar to class IIa), GarvieacinQ (#33) (class IIa), Bovicin255 (#42) (class IId), Bovicin255 peptide (#46) (class IId), and Bacteroidetocin A (#22) (similar to class IIa) also displayed differences in their species inhibition patterns. For instance, Bovicin255 peptide (#46) specifically targeted the Gram-negative species *Bacteroides fragilis DSM2151* and *Collinsella aerofaciens DSM3979*. In contrast, its closely related sister sequence, Bovicin255 (#42), did not exhibit inhibition against these two species. This highlights that even slight variations in the sequence can lead to differences in the target spectrum of the bacteriocins, a tendency generally observed for class IIa bacteriocins.^50^

To assess the therapeutic potential of these bacteriocins as novel antibiotics or precise therapeutics, we scrutinized their efficacy against gut microbes implicated in health disorders. One strain: *Collinsella aerofaciens*, a biosafety level 2 organism has been implicated in psoriasis^51^ and coronary artery disease (CAD),^52^ yet remains highly abundant in the general population, detected in 86% of the 396 stool samples analyzed in the Human Microbiome Project.^31^ Our study revealed that Actifencin (#13) and Bovicin255 peptide (#46) effectively target this strain. The targeted action of Bovicin255 peptide (#46) against *Collinsella aerofaciens* underscores its potential for selectively eliminating harmful species without significantly disturbing the inherent ecology of the gut microbiota, given its specificity to only five species within our gut microbiota collection.

Another example involves *Eubacterium rectale* DSM 17629, identified as a ‘driver’ bacterium that plays a role in cancer initiation by fostering inflammation.^53^ This strain is uniquely susceptible to Actifencin (#13), positioning this bacteriocin as a promising candidate for therapeutic interventions aimed at reducing inflammation. Additionally, targeting *Bacteroides vulgatus* merits consideration, given that certain strains of this species have been implicated in exacerbating colitis.^54^

### Selective modification of fecal-derived microbial communities ex vivo

In light of the results from our *in vitro* screenings, we postulated the possibility of specifically targeting and eliminating particular species within complex microbial communities. Our objective was not only to corroborate our *in vitro* observations but also to investigate the emergence of novel inhibitory patterns attributable to the bacteriocins under study. To this end, we selected three bacteriocins, each exemplifying distinct inhibitory profiles, to represent the breadth of functionality within our bacteriocin collection. The chosen bacteriocins were as follows: Ubericin A (#12) for its selective activity against Gram-positive bacteria; Actifencin (#13), noted for its wider spectrum of action encompassing both Gram-positive and Gram-negative bacteria; and Bacteroidetocin A (#22), distinguished by its specific efficacy against Gram-negative bacteria. To assess the effect of the selected bacteriocins, these were heterologously produced, filtered and mixed with agar to create bacteriocin containing agar plates.

### Three distinct base media were selected for the plate design

1. mGAM, favored for its capacity to support broad cultivation of gut microbiota species.^30^ 2. mGAM supplemented with 5 µg/ml of vancomycin and 50 µg/ml of kanamycin, known to facilitate the selective growth of Bacteroides species.^55,56^ This was particularly chosen to elucidate the inhibitory effects of Bacteroidetocin A (#22), reflecting its targeted spectrum against Bacteroides species 3. MRS specifically employed to examine the interactions between lactic acid bacteria and the bacteriocins Ubericin A (#12) and Actifencin (#13).^57^

**Figure 2.**
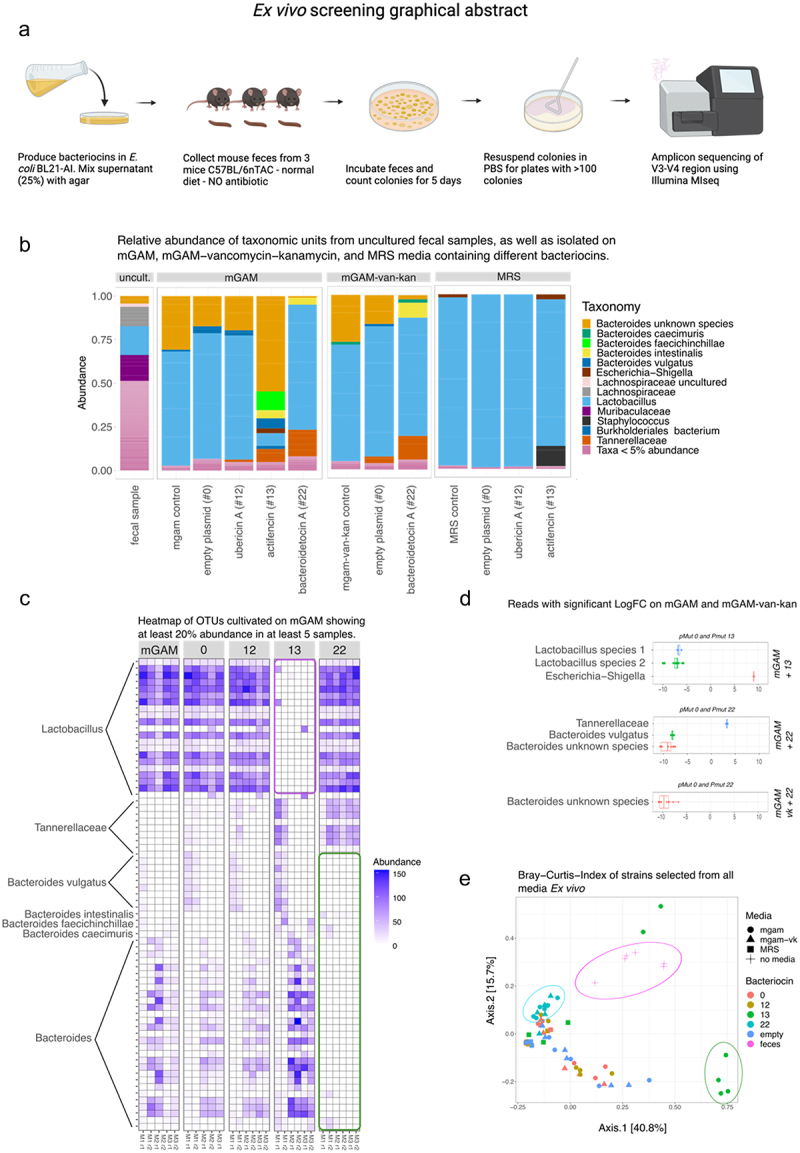
(a) Graphical abstract of the ex vivo experimental setup. (b) Relative abundance plot for strains isolated on mGAM mixed water (control) or the bacteriocins 0, 12, 13 and 22, mGAM-vancomycin-kanamycin mixed water (control) and with the bacteriocins 0 and 22, and MRS mixed with water (control) or the bacteriocins 0, 12, and 13. (c) Heatmap showing the reads assigned from colonies isolated from mGAM mixed with water (control) or the bacteriocins 0, 12, 13 and 22, having at least 20% abundance in 5 samples. Notably the abundance of Lactobacillus is almost zero on the mGAM media mixed with supernatant from Actifencin (#13) (purple square), whereas the abundance of Bacteroides is clearly enriched. Bacteroides species on the other hand is depleted on the mGAM media mixed with supernatant from Bacteroidetocin a (#22) (green square). (d) Significant features found on the selection plates: mGAM + actifencin (#13), mGAM + bacteroidetocin a (#22), and mGAM-vancomycin+kanamycin + bacteroidetocin a (#22). Significance is based on LogFC compared to control plates. (Significance level, padj < 0.05). Negative values indicate less abundance on the bacteriocin plates compared to control. (All tests were made with DAtest and EdgeR - Quasi likelihood test). (e) Beta-diversity plot showing that some samples group together based on media and bacteriocin selection criteria.

Three independent murine fecal samples were serial diluted, plated, and incubated for 5 d on the bacteriocin + media selection plates. Using 16S rRNA amplicon sequencing we characterized the microbial community composition after cultivation for the different bacteriocin and media combinations (Figure 2a).

Relative abundance plots were generated to analyze the distribution of reads across different media and bacteriocin conditions. The plots revealed distinct patterns of reads mapped to specific genera. On the mGAM media, the majority of reads were associated with the genus *Lactobacillus*, except in the mgam + actifencin (#13) condition where a higher abundance of reads mapped to *Bacteroides* was observed (Figure 2b). This difference was found to be statistically significant (Figure 2d), with a significant decrease in an unclassified *Lactobacillus* species and the overall genus *Lactobacillus*. Concurrently, an increase in the *Escherichia-Shigella* genus was observed (Figure 2d).

On the MRS selection plates, reads primarily mapped to the genus *Lactobacillus*, but no significant differences were observed among the different groups (Figure 2b). Most likely this was due to the selectivity of MRS media toward *Lactobacillus* in general. In the case of the mGAM + bacteroidetocin A (#22) selection plates, a significant decrease in *Bacteroides* and specifically *Bacteroides vulgatus* was observed (Figure 2b,d). These results indicate that Bacteroidetocin A (#22) is indeed capable of inhibiting naturally occurring microbiota strains of *Bacteroides vulgatus* in a complex community. The relatively high abundance of *Lactobacilli* on the mGAM-vk-kan plates can be explained by these species inherent resistance toward these drugs^58^ A heatmap was generated to visualize the abundance of species selected on the selection plates.

The results showed a clear reduction of reads assigned to the genus *Lactobacillus* selected on the media mGAM + actifencin (#13), but not on MRS + actifencin (#13) media. Additionally, there appeared to be a decrease in reads mapped to the genus *Bacteroides*, particularly *Bacteroides vulgatus*, on the mGAM/mGAM-vk + bacteroidetocin (#22) selection plates (Figure 2C). Beta-diversity analysis of these samples revealed distinct clusters corresponding to the selection media and the different bacteriocins used. Uncultured fecal samples are clustering together, as well reads assigned from the mGAM + actifencin (#13) media. Reads assigned from the mGAM/mGAM-vk + bacteroidetocin (#22) likewise clusters together on the heatmap. This visualization of the clustering backs up the findings also shown in the abundance and heatmap figures (Figure 2e).

### *Evaluation of heterologously expressed bacteriocins from* E. coli *Nissle strains in mice*

To further investigate the functional impact of the heterologously expressed bacteriocins in a physiological context, Actifencin (#13) and Bacteroidetocin A (#22) were selected as candidates for *in vivo* experimentation, motivated by their pronounced ability to modulate the microbiota in *ex vivo* settings. For this purpose, we engineered the probiotic strain *Escherichia coli* Nissle *(EcN)*. *EcN* was selected due to its favorable characteristics as a safe probiotic organism (GRAS status) and its robust growth capabilities under anaerobic conditions.^40^ For this purpose, we utilized the native *EcN* plasmid pMUT1, which had previously been employed in our *in vitro* and *ex vivo* assays. To ensure consistent bacteriocin production in the host it was modified to contain a constitutive promoter to drive bacteriocin expression, replacing the originally inducible promoter.

To ensure the suitability of the engineered *EcN* strains, we evaluated their growth rates and confirmed their functional properties through an overlay spot assay against indicator strains. The engineered strains *EcN_*pMUT13_actifencin and *EcN_*pMUT22_bacteroidetocinA exhibited an average doubling time that was 50% and 37.5% higher than that of the *EcN_*WT_GFP strain. The *EcN_*pMUT0_control strain showed an increase in doubling time of 15% (Supplementary Figure S1).

**Figure 3.**
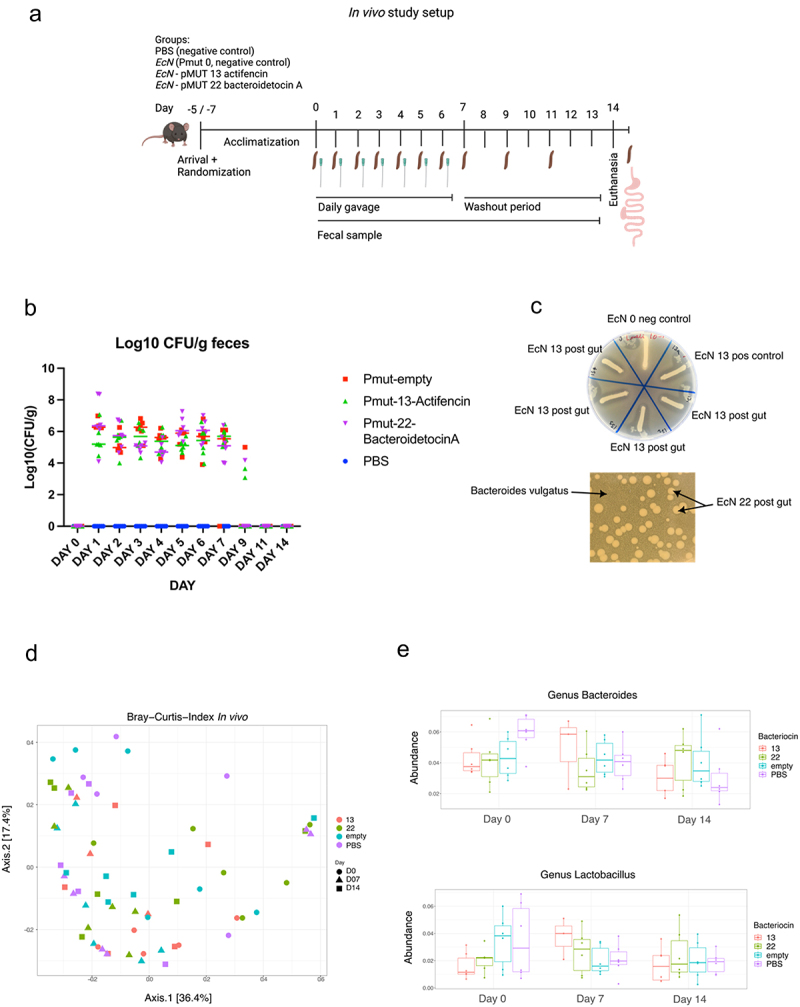
(a) graphical representation of the in vivo study showing the gavage and fecal collection. (b) Log10 (Cfu/g feces) of surviving EcN in the feces after 24 h post gavage. (c) Overlay spot assay of EcN isolated from fecal samples of mice orally dosed with EcN_pmut13_actifencin and EcN_pMUT22_bacteroidetocinA. Inhibition zones can be visualized around the growing cultures. EcN 0 refers to the strain producing no bacteriocin, EcN 13 refers to the lab strain, EcN 13.A-d refers to individual colonies re-streaked on LB plates from the fecal samples. Indicator strain used: L. mali DSM 20,444. Bacteroidetocin a (#22) was assessed directly from the plating of feces on selection plates using top agar containing B. vulgatus. (d) Beta-diversity plots showing no clear patterns of clustering between the groups, as opposed to what was found in the ex vivo study. (e) Relative abundance plots shown for the genus: Lactobacillus and Bacteroides for the study days: 0, 7 and 14. No significant difference was to be found between the different days for these two genera between the groups.

The *in vivo* study spanned a period of 7 consecutive days, during which the mice received daily oral doses of the respective strains followed by a 7-day washout period. This study design allowed us to investigate the colonization potential of the bacteriocin-producing strains compared to the control group and confirm the elimination of the *EcN* strains from the gut over time (Figure 3a). Colonization of the *EcN* cultures was seen to be stable during the days of gavage: approximately 10^5^-10^6^ colony-forming units (CFUs) per gram of feces over the 7-d oral delivery period. By day 9, the CFUs decreased to approximately 10^4^, and no surviving *EcN* colonies could be detected on days 11 or 14 (Figure 3b).

The functionality of *EcN* strains post transit the murine gut from fecal samples was confirmed using an overlay spot assay with indicator strains *L. mali DSM 20444* and *B. vulgatus DSM 1447*. Detection of inhibitory zones around colonies validated the bacteriocin production of *EcN_*pMUT22_bacteroidetocinA and re-streaks of *EcN_*pMUT13_actifencin from the fecal samples were compared to the *EcN* strain before entering the mouse gut as well as a negative *EcN_*pMUT0 control, showing distinct inhibition zones around the four *EcN* colonies tested from the fecal samples (Figure 3c). Nine colonies of both *EcN*_pMUT13_actifencin and *EcN*_pMUT22_bacteroidetocinA underwent colony PCR and Sanger sequencing, confirming their identity as the correct *EcN* strains after having been through the murine gut with no mutations detected in the bacteriocin gene or promoter region.

Fecal samples were collected on study day 0 (prior to the first oral delivery), day 7, and day 14 for sequencing analysis. Despite the *ex vivo* findings indicating differential abundance of certain bacterial genera and species, no distinct difference emerged between the experimental groups receiving gavage with *EcN* producing bacteriocins, and the control group treated with PBS (Figure 3e). Specifically, we focused on examining the overall abundance of *Lactobacillus* and *Bacteroides vulgatus*, since these species exhibited differential abundance in the *ex vivo* study. Likewise, beta-diversity plots showed no distinct patterns of difference between the groups, indicating that no discernable difference was to be found between the groups (Figure 3d).

## Discussion

Bacteriocins and their native producers have been extensively used in the food industry to combat food pathogens. Recent studies have explored their potential as novel antimicrobial agents, particularly against antibiotic-resistant pathogens. Their potential and benefits as a novel group of antibiotics are highlighted by their narrow specificity compared to traditional antibiotics, as well as their ability to not disturb the overall composition of the microbiota.^4,8,14^

In our research, we set out to establish a robust, high-throughput methodology for heterologous expression and production of class II bacteriocins. We then applied this extensive bacteriocin collection to assess their impact on a carefully selected array of gut microbiota strains. Utilizing this platform, we conducted a comprehensive *in vitro* screening, testing 75 class II bacteriocins against 48 species, including pathogens like *Clostridium difficile* and *Salmonella enterica*.

Consistent with previous studies, most of the class II bacteriocins we expressed, generally did not affect Gram-negative species.^4,13^ The exceptions were Actifencin (#13) and Bacteroidetocin A (#22). These bacteriocins display traits akin to class II bacteriocins, yet they are not categorized within this classification. These two bacteriocins likewise showed an ability to selectively suppress species in our *ex vivo* study probably due to their greater target spectrum. Actifencin (#13) and Bacteroidetocin A (#22) suppressed *Lactobacillus* and *Bacteroides* genera, respectively, while largely preserving the diversity of other species of the microbiota.

Actifencin, natively produced by *Actinomyces ruminicola DPC 7226*, has been characterized by Sugrue et al.,^23^ and is believed to be part of a new class of bacteriocins produced by the *Actinomyces* genus. Sugure et al.^23^ observed that 47 out of 161 *Actinomyces* genomes contained at least one Actifencin-related bacteriocin gene, showcasing significant sequence diversity. Our study indicates that this group of bacteriocins holds significant promise as antimicrobials, a potential further highlighted by their attribute of requiring only the bacteriocin gene for expression, akin to other class II bacteriocins. One application could be to target LAB species, commonly known for their probiotic benefits, but which have also been implicated in rare infections, particularly among diabetic and immunocompromised individuals^59^ and have been associated with increased microbial ethanol production contributing to nonalcoholic fatty liver disease (NAFLD).^60,61^ Therefore, identifying bacteriocins that targets specifically this group of gut bacteria, could prove beneficial.

Our study also sheds light on bacteroidetocin A (#22), a distinctive bacteriocin initially characterized by Coyne et al.^24^ and natively produced by *Bacteroides vulgatus*. Our analysis discerned the ability of Bacteroidetocin A to impede four species: *Prevotella copri DSM 18205*, *Bacteroides vulgatus DSM 1447*, *Bacteroides clarus DSM 22519*, and *Bacteroides stercoris DSM 19555*. Further bolstering these findings, our *ex vivo* sequencing analysis demonstrated a significant reduction in reads attributed to the *Bacteroides* genus, including *Bacteroides vulgatus* itself. Interestingly, we observed an increase in reads associated with the *Tannerellaceae* family within the *Bacteroidales* order, suggesting a selective inhibitory action of Bacteroidetocin A, where not all *Bacteroides* strains are susceptible to its effects.

Considering the strain-dependent involvement of *B. vulgatus* in colitis, as indicated by Li et al.^54^ underscores the critical need to expand our understanding of the specificity with which Bacteroidetocin A targets different strains. Despite its demonstrated antibacterial effects, the precise mechanism by which Bacteroidetocin A operates remains elusive.^24^ This gap in knowledge accentuates the imperative for ongoing research to not only unravel the action of Bacteroidetocin A but also to understand its full antimicrobial potential effectively.

In our study, we compiled a comprehensive list of species inhibited by at least one of the expressed bacteriocins, which facilitated the identification of species selectively targeted by only a few bacteriocins. This approach revealed previously undocumented inhibitory relationships. Notably, *Collinsella aerofaciens* DSM 3979, known to proliferate in patients with psoriasis and coronary artery disease, was found to be selectively inhibited by Actifencin (#13) and Bovicin 255 (#46). Remarkably, Bovicin 255 (#46) demonstrated inhibition on only three additional species, positioning it as a prime candidate for targeted intervention in the gut microbiota without disrupting the overall gut ecosystem. These findings underscore the potential of specific bacteriocins, to not only modulate disease-associated microbial populations but also to offer targeted strategies for mitigating inflammation and other disease processes.

Having an extensive catalog of bacteriocins and their specific inhibition patterns is invaluable in the search for novel antibiotic candidates. By understanding which bacteriocins target specific species, we can more effectively employ these agents in therapies designed to preserve the balance of the gut microbiota while combating pathogenic strains.

In our pursuit to understand if Actifencin (#13) and Bacteroidetocin A (#22) capable of altering mouse fecal microbiota *ex vivo* could also effect change *in vivo*, we engineered a probiotic *EcN* strain for heterologous expression. The utilization of a heterologous host like *EcN* offers a strategic advantage over relying on natural bacteriocin producers, circumventing numerous challenges related to colonization, safety, expression patterns, and yield. Moreover, it presents a more viable alternative to purified bacteriocin delivery, which is often hampered by rapid degradation within the intestinal tract and the high costs associated with production and purification.

Despite the bacteriocin-producing *EcN* strains retaining their ability to produce bacteriocins after passage through the murine gut, we observed no significant differences in the gut microbiota composition between mice treated with these strains and the control groups. One potential reason for the observed lack of significant gut microbiota shifts could include suboptimal bacteriocin production. Another reason could be the differences of gut colonization by LABs and *EcN*. LABs predominantly inhabit the upper intestine^62^ whereas *EcN* is more prevalent in the colon.^63^ Additionally, the tendency of *B. vulgatus* and other *Bacteroides* members to form biofilms and colonize the intestinal mucosa,^64^ presents additional challenges in bacteriocin application. These discordances highlight the nuanced and context-sensitive nature of bacteriocin activity, illustrating the difficulties in translating *in vitro* findings to *in vivo* contexts.

Recently, Mortzfeld et al.^20^ engineered *EcN* to produce Microcin I47 to target *K. pneumonia* in mice. The authors found a reduction in *K. pneumonia* compared to an *EcN* control. Despite the usage of antibiotics to eliminate the effects of the native microbiota prior to *K. pneumonia* and *EcN* delivery, and consequently a disturbance of the native microbiota pre- and post-treatment, the authors did not find any significant difference in the microbiome composition post-treatment between *EcN* and PBS control group. Such findings indicate that bacteriocin delivery through probiotic strains holds promise as a future therapy without disruption of the native microbiota. This strategy of pre-treating mice with antibiotics, could pose a potential strategy to minimize the background noise created by the presence of a complex microbiota and amplify the signal from both the producer and target strains when performing metagenomic sequence analysis.

A discrepancy between *in vitro* and *in vivo* findings is a commonly observed phenomenon in bacteriocin research. For example, Dobson et al.^12^ reported that while Lacticin 3147 produced by *Lactococcus lactis* DPC6520 was effective *in vitro* against *Listeria monocytogenes*, it failed to show inhibitory effects in a mouse model, despite the survival and functional persistence of *L. lactis* in the gastrointestinal tract.^12^ To strengthen the effectiveness of bacteriocin delivery *in vivo*, strategic optimizations are required. One strategy, as demonstrated by Field et al.^5^ involves enhancing protein stability while retaining bacteriocin efficacy of nisin. Alternatively, choosing a production host that aligns with the specific gut locations of target strains, for instance, by using *Lactococcus lactis*^65^ or *Lactobacillus plantarum*^66^ as host organisms to facilitate more effective inhibition of LAB species.

Our research – spanning *in vitro*, *ex vivo*, and *in vivo* studies – underscores the significant potential of bacteriocins as precise modulators of gut microbiota, showing selective inhibition in complex gut microbiota samples. Despite the complexities of *in vivo* activity, our study describes a method to evaluate the potential of bacteriocins in a complex microbiota using *ex vivo* and *in vitro* methods, serving as an important starting point in describing and finding novel bacteriocin candidates and opens up avenues for future exploration and application in microbial therapeutics.

## Data Availability

The datasets supporting the conclusions of this article are available in the NCBI repository. *Ex vivo* data: BioProject: PRJNA1007563.^67^ The *In vivo* dataset supporting the conclusions of this article are available in the NCBI repository: BioProject ID: PRJNA1007568.^68^ The datasets supporting the conclusions of this article are included within the article and its additional files.
